# ^1^H NMR Metabolomics on Pigs’ Liver Exposed to Antibiotics Administration: An Explorative Study

**DOI:** 10.3390/foods12234259

**Published:** 2023-11-25

**Authors:** Maria Pia Fabrile, Sergio Ghidini, Augusta Caligiani, Federico Scali, Maria Olga Varrà, Veronica Lolli, Giovanni Loris Alborali, Adriana Ianieri, Emanuela Zanardi

**Affiliations:** 1 Department of Food and Drug, University of Parma, Strada del Taglio, 10, 43126 Parma, Italy; mariapia.fabrile@unipr.it (M.P.F.); sergio.ghidini@unipr.it (S.G.); augusta.caligiani@unipr.it (A.C.); mariaolga.varra@unipr.it (M.O.V.); veronica.lolli@unipr.it (V.L.); adriana.ianieri@unipr.it (A.I.); 2 Istituto Zooprofilattico Sperimentale della Lombardia e dell’Emilia-Romagna, Via A. Bianchi 9, 25124 Brescia, Italy; federico.scali@izsler.it (F.S.); giovanni.alborali@izsler.it (G.L.A.)

**Keywords:** antibiotic-free claim, untargeted metabolomics, NMR, pork, polar metabolites, non-polar metabolites

## Abstract

An untargeted Nuclear Magnetic Resonance (NMR) spectroscopy-based metabolomics approach was applied as a first attempt to explore the metabolome of pigs treated with antibiotics. The final goal was to investigate the possibility of discriminating between antibiotic-treated (TX group) and untreated pigs (CTRL group), with the further perspective of identifying the authentication tools for antibiotic-free pork supply chains. In particular, 41 samples of pig liver were subjected to a biphasic extraction to recover both the polar and the non-polar metabolites, and the ^1^H NMR spectroscopy analysis was performed on the two separate extracts. Unsupervised (principal component analysis) and supervised (orthogonal partial least squares discriminant analysis) multivariate statistical analysis of ^1^H NMR spectra data in the range 0–9 ppm provided metabolomic fingerprinting useful for the discrimination of pig livers based on the antibiotic treatment to which they were exposed. Moreover, within the signature patterns, significant discriminating metabolites were identified among carbohydrates, choline and derivatives, amino acids and some lipid-class molecules. The encouraging findings of this exploratory study showed the feasibility of the untargeted metabolomic approach as a novel strategy in the authentication framework of pork supply chains and open a new horizon for a more in-depth investigation.

## 1. Introduction

Today, the meat sector faces many challenges in terms of sustainability, nutritional aspects and authenticity, leading meat operators to rethink the management of food-producing husbandry systems. Additionally, as consumers incline toward making choices, which are seen as greener and more sustainable, greater attention is devoted to the positioning factors of meats in the market [1]. Currently, several certifications and labels guarantee various meat attributes, including antibiotic-free, halal, kosher, organic and GMO-free [2]. Certification marks concerning the usage of antimicrobials (AMU) (i.e., *Antibiotic Free* or *Raised Without Antibiotics*) fulfill consumer requests concerning value-added meat obtained from farm animal chains embracing animal health and welfare values [3]. On the other hand, responsible AMU in livestock farming responds to the global need to fight antimicrobial resistance (AMR)—a major threat to public health [4]. The definition of novel attributes in meat may provide opportunities for food fraud, and for this reason, fit-for-purpose tools are urgently needed for the protection of the integrity of the meat chain.

For public health protection, in the European Union (EU), the procedures establishing the maximum concentration of a residue of pharmacologically active substances, which may be permitted in food of animal origin, are laid down by Regulation (EC) n. 470/2009, and the maximum residue limits (MRLs) of those substances in foodstuffs of animal origin are set out in Commission Regulation (EU) n. 37/2010 [5,6]. The regulatory framework for the manufacturing, import, export, supply, distribution, pharmaco-surveillance, control and the use of veterinary medicinal products and medicated feed is represented by Regulation (EU) 2019/6 and Regulation (EU) 2019/4 [7,8]. Among the objectives, these provisions, together with further implementing and delegated regulations, aim at strengthening EU action against AMR through specific measures, such as prudent AMU and the reservation of certain antibiotics only for the treatment of infections in human medicine. For monitoring purposes, all member states must include official controls in the multi-annual national control plans (MANCPs) with regard to antimicrobial agent residues in animals and derived food; in particular, the specific requirements, criteria and uniform practical arrangements for the performance of official controls of residues are provided by Commission Delegated Regulation (EU) 2022/1644 and Commission Implementing Regulation (EU) 2022/1646 [9,10].

Concerning the analytical aspects in relation to residues of pharmacologically active substances in live food-producing animals and products of animal origin, the provisions applicable to the methods used for sampling, laboratory analyses and interpretation of analytical results are established by Commission Implementing Regulation (EU) 2021/808, which applies to official controls aimed at verifying compliance with MRLs [11]. In this framework, biological and biochemical methods are available mainly for screening purposes [12], while chromatographic analysis based on full-scan diode array detection spectrophotometry, fluorescence detection spectrophotometry or mass spectrometry detection is required as a confirmatory method, except for prohibited or unauthorized pharmacologically active substances, for which only mass spectrometry detection is suitable. By providing full or complementary information to unequivocally identify the substance, confirmatory methods for antibiotic residue monitoring are based on targeted multiresidue methods limited to a pre-defined number of known analytes [13,14]; the literature provides several examples of validated multiresidue multiclass methods, which cover more than one hundred targeted analytes in a single analysis [15,16]. However, the untargeted approach has emerged as an alternative and promising tool enabling the development of sensitive and wide-ranging screening of analytes, overcoming the limitations of targeted analysis. The untargeted workflow allows for the recovery of an enormous number of non-pre-selected compounds, even unknown ones, which may be simultaneously monitored and identified through a bottom-up strategy [17]; the screening of thousands of molecules may highlight new analytes as potential biomarkers of the physiological responses following the administration of antibiotics. In a long-term perspective, novel biomarkers might be a useful strategy for implementing more efficient control systems enabling the monitoring of a more representative number of samples within the residue control plans. As a matter of fact, the report of the European Food Safety Authority for 2021 on the results from the monitoring of veterinary medicinal product residues in live animals and animal products reveals a continuous decline in the percentage of non-compliant samples over the last decade [18]. Despite the positive trend from a safety point of view, the very low non-compliance percentage (0.14% for antibacterials for all tested animal species, 0.09% for pigs in 2021) could be affected by the inadequacy of sampling and may highlight the need for new approaches. 

Apart from compliance purposes for MRLs, the untargeted approach could play a role in assessing the specific quality certifications related to the use of antibiotics in animal husbandry beyond the presence or absence of residues in the samples under investigation. Changes in gut-microbiota-produced metabolites after antibiotic exposure in pigs are documented in the literature [19,20]. Moreover, there is evidence that antibiotic treatment elicits microbiota-independent changes in host metabolites [21], markedly affecting the metabolomic profiles of the gut and biofluids in antibiotic-treated piglets [22]. In this respect, it is conceivable that the metabolome—intended as a whole set of precursors, intermediates and products of biochemical processes—may contain useful information for revealing the metabolites attributable to an antibiotic treatment.

Metabolomics is the comprehensive study of low-molecular-weight (typically <1500 Da) metabolites in biological systems. In recent years, this high-throughput molecular technology has been widely exploited to study several traits of interest in animal sciences. It has been explored as an efficient methodology for unraveling metabolic changes in food-producing animals, thus helping identify the candidate molecules representative of different physiological pathways and contributing to the development of diagnostic tools for better animal management [23]. In this regard, the advances in metabolomic profiling have led to an investigation of the effects of heat stress on the saliva and serum metabolome of pigs and identification of metabolites, which could be used as biomarkers of the stressful condition [24,25]. Metabolomics has been proposed as a promising tool for investigating novel biomarkers useful for a comprehensive assessment of animal welfare *status*; objective indicators of the overall *status* of the animal might be advantageous for defining adequate management strategies to improve the welfare of livestock animals and for the purpose of authenticity and traceability of meat supply chains, which adopt high standards of production and label claims concerning animal husbandry conditions [26]. Two main analytical platforms—namely nuclear magnetic resonance (NMR) spectroscopy and mass spectrometry (MS)—are considered the workhorses for metabolomics analysis, leading to the generation of a massive amount of data due to the acquisition of thousands of metabolite signals [27,28]. 

To the best of the authors’ knowledge, insights into the biochemical phenotype through the study of the metabolome of pigs treated with antibiotics have not been reported previously. Therefore, the aim of the present study was to explore the metabolome via an untargeted NMR-based approach in order to capture and compare the metabolomic fingerprinting of antibiotic-treated vs. untreated pigs. This study is intended as a first step of a wider investigation aimed at identifying and validating adequate biomarkers to be used as tools for assessing the pig chains’ authenticity. In this regard, the pigs in this study were from commercial farms selected within the ClassyFarm system—the Italian national monitoring system aimed at categorizing livestock farms based on risk. 

## 2. Materials and Methods

### 2.1. Chemicals and Reagents

HPLC grade methanol, chloroform and water were purchased from Labscan (Dublin, Ireland). Analytical grade sodium dihydrogen phosphate monohydrate and di-sodium hydrogen phosphate dihydrate were supplied by Merck (Darmstadt, Germany). Deuterium oxide (99.9% D), methanol-d_4_ (99.8% D) and chloroform-d_4_ (99.8% D) were obtained from VWR International BVBA (Geldenaakseban, Leuven, Belgium), and 3-(trimethylsilyl)-propionate-d_4_ (TSP) was obtained from Sigma-Aldrich (Milano, Italy).

### 2.2. Experimental Design

**Figure 1:**
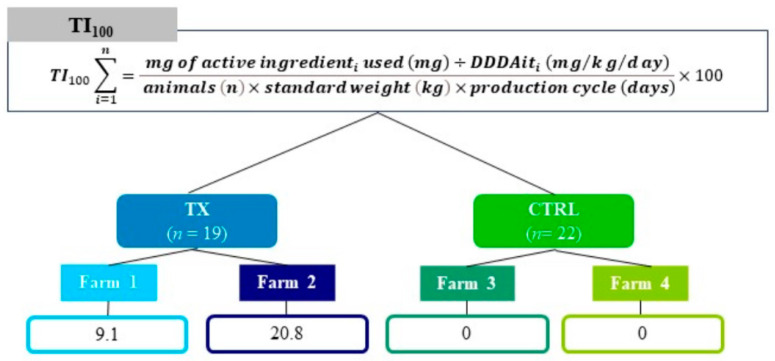
Graphical representation of the experimental design. The upper box sums up the mathematical formula for TI_100_ used as classification criterion for assigning group ID (CTRL and TX). (Active ingredient used (mg): amount of the active ingredient of the antibiotic used for treatment; DDDAit (mg/kg/day): Defined Daily Dose Animal for Italy expressed in mg of active substance per day per kg of body weight; Animals (*n*): number of animals considered for batch of livestock; Standard weight (kg): average animal weight during the treatment period, set up at 100 kg for heavy pigs; Production cycle (days): days of pig production cycle).

Forty-one heavy pigs (approximately 170 kg of live body weight) reared in 2020 on four fattening farms in northern Italy were selected for this study. Pigs were randomly allotted into two groups according to the object under investigation: the control (group ID CTRL, *n* = 22) and treatment (group ID TX, *n* = 19) groups, respectively. Group classification was designed considering the AMU, which was estimated by calculating treatment incidence 100 (TI_100_) using the Defined Daily Dose Animal for Italy (DDDAit) as a standard, as described in a previous study on Italian fattening farms [29]. The TI_100_ can be interpreted as the percentage of time a pig spent under treatment during its production cycle [30]. Data on AMU were extracted from the Italian Ministry of Health’s surveillance system, ClassyFarm [31], which is available and mandatory for all Italian pig farms. In this study, for greater representativeness of the samples, pigs in the TX group were randomly chosen from two batches of about 100 pigs each from two farms, namely Farm 1, with a TI_100_ of 9.1, and Farm 2, with a TI_100_ of 20.8; similarly, pigs in the CTRL group were chosen from two batches from Farm 3 and 4, respectively, where no antibiotic treatments were recorded (Figure 1). 

Except for antibiotic administration, other variables, such as feed and gender, were intentionally not considered to assure greater reliability of the study. However, all pigs were of the same age and were slaughtered on the same day at a commercial abattoir under the supervision of the veterinary team and were intended for human consumption. No ethical approval was required. The whole liver of all pigs was removed from the carcasses during slaughtering and immediately stored at −20 °C until analysis. The liver was chosen as the matrix for the investigation, since its role in drug metabolism is well established. 

### 2.3. Sample Preparation

The biphasic extraction procedure, known as the Bligh and Dyer method [32], with slight modification was adopted for the recovery of polar and non-polar metabolites of the liver. Briefly, 100 mg of frozen liver manually grinded was mixed with 3 mL mixture of methanol/chloroform (2:1, *v*/*v*) in a 15 mL screw cap glass tube and vortexed for 30 s. Then, a sonication step in an ice-water bath for 30 min was performed. Further, 1 mL of water and 1 mL of chloroform were added and newly vortexed. The sample was then centrifuged for 35 min at 4 °C, at a speed of 2220× *g* (Beckman Coulter Life Sciences, Milano, Italy). Finally, phase separation was achieved, and the upper polar and lower non-polar fractions were separately transferred to new glass tubes, and each fraction was dried under nitrogen flow. Both fractions were stored at −20° C and reconstituted prior to ^1^H NMR analyses. For the polar extracts, 700 µL of sodium phosphate buffer in D_2_O (0.25 M; pH = 7) and 100 µL of TSP as internal standard were added and transferred in 5 mm outer diameter NMR tubes. For the non-polar extracts, 600 µL of chloroform-d_4_ and 200 µL of methanol-d_4_ were employed for the reconstitution. 

### 2.4. ^1^H NMR Spectroscopy Analysis

Polar and non-polar extracts were separately recorded using NMR spectrometer operating at a magnetic field of 600.17 MHz with a 5 mm ROYAL probe (JEOL ECZ 600, JEOL Ltd., Tokyo, Japan). For both extracts, each acquisition was preceded by a shimming phase along the x-, x/z-, y- and y/z- axes performed at 25 °C (temperature delay, 120 s). Different setting conditions were applied with the Delta software package (ver. 5.3) to optimize the quality of spectra acquisition, as follows. For the polar extracts, proton acquisition was performed at 298 K, with a field frequency lock on D_2_O, 32,768 data points using a 30° pulse length and 5 s of relaxation delay. A total of 128 scans were collected over a spectral width of 24 ppm. The polar aqueous spectrum was collected by employing the basic shape pulse obs_DANTE_presaturation (JEOL ECZ600R database) for the suppression of water signal in the spectral region between 4.5 and 4.8 ppm (δ = 4.661 ppm). For the non-polar extracts, proton acquisition was conducted at 298 K, with a field frequency lock on methanol-d_4_, 65,536 data points using 30° pulse length and 10 s of relaxation delay. A total of 32 scans were collected over a spectral width of 24 ppm. 

### 2.5. Data Processing and Multivariate and Univariate Data Analysis

Raw spectra were transferred to the MestReNova 14.2.1 software (Escondido, CA, USA) to manually perform the correction for the phase and baseline. With regard to the polar fraction, all spectra were referenced to TSP (δ = 0 ppm). For non-polar fraction, δ = 0 ppm allowed a perfect alignment of the spectra; therefore, it was also used as the referencing signal. The assignment of ^1^H NMR signals was supported by the literature [33,34,35,36,37,38,39] and online public databases for NMR, such as the Biological Magnetic Resonance Data Bank (BMRB) [40] and the Human Metabolome Data Base (HMDB) [41]. The integration step was manually performed on all fully overlapped spectra to identify and consider only the interesting region; thus, the integration pattern was built considering the region between 0 and 9 ppm for both polar and non-polar fractions. Spectral regions containing resonances only from noise, water and TSP were excluded prior to data analysis. To compensate for the concentration differences, each integral region was normalized to the TSP signal area for the polar fraction or to the total area of each spectrum in the case of non-polar fraction. Finally, the metabolomics dataset was built as a data matrix N × R [N = samples; R = ppm buckets], leading to the acquisition of a 41 × 84 (3444) and a 41 × 76 (3116) data matrix for polar and non-polar fractions, respectively. No missing data were detected in the two matrices. The two datasets were exported to the SIMCA 17 software package (v. 17.0.0, Sartorius Stedim Data Analytics AB, Umea, Sweden), and statistical analysis was performed separately on the two different fractions. Unsupervised principal component analysis (PCA) and supervised orthogonal partial least squares discriminant analysis (OPLS-DA) were performed for the different objectives they pursue. PCA aims at exploring the natural distribution of the samples within a reduced dimensional space and looking for clustering trends among samples; the PCA score plots facilitate data projection from a higher dimensional space to a lower dimensional one and enable reconstruction of them without prior assumptions regarding their distribution. On the contrary, the OPLS-DA is a discriminant and classification method; it usually provides superior classification results to the PCA (which is not a classification method) because it focuses on the boundaries separating the pre-defined groups in the multidimensional space. Moreover, taking advantage of variable selection methods, OPLS-DA supports the identification of possible biomarkers, thus enhancing the interpretability of the datasets. In the present study, PCA was preliminarily performed on the auto-scaled datasets to explore their characteristics and detect clustering or trends among the samples. The presence of outliers was also checked by evaluating Hotelling’s T^2^ range values (5% level of significance). The PCA models were internally validated with a 7-fold cross-validation (CV), and their quality was assessed by the performance indicators goodness-of-fit (R^2^X) and predictive ability (Q^2^). To further explore the datasets, the OPLS-DA was performed on the two matrices after Pareto scaling of the data; internal validation of the computed models was carried out with a 7-fold CV, and the R^2^X, Q^2^ and R^2^Y (the fraction of Y variation modelled in that component) values were considered as useful performance indicators for evaluating the global quality of the models. Finally, the variable importance in projection (VIP) scores for OPLS-DA components were designed to find the strongest influence exerted by NMR signals over sample discrimination: as a rule, VIP values ≥ 1 were used to identify the most relevant buckets (selected features), i.e., those with the largest discriminatory power, since scores smaller than one indicate a non-important variable for discrimination between groups [42]. The OriginPro 2019 software (OriginLab Corporation, Northhampton, MA, USA) was used for univariate data analysis. To check the normality of the selected features, the Shapiro–Wilk test was conducted. The non-parametric Mann–Whitney U-test (*p* ≤ 0.05) and the two-sample *t*-test (*p* ≤ 0.05) were applied to the selected features not fitting and fitting the normal distribution, respectively, to investigate the differences between the two groups—TX vs. CTRL. The fold change (FC) ratio was calculated by taking the median value of selected features in the TX group over that of the CTRL group to highlight the metabolites’ accumulation, where <1 = down-accumulated and <1 = up-accumulated [43]. 

## 3. Results

### 3.1. Assignment of ^1^H NMR Spectra Signals of Hepatic Polar and Non-Polar Extracts

**Figure 2:**
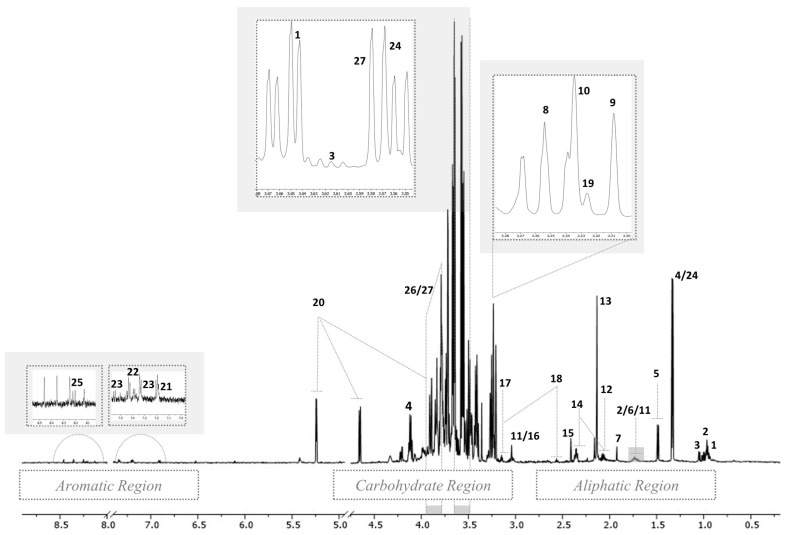
Representative 600 MHz 1D ^1^H NMR spectrum of the polar extract of pig liver. For the signal numbering (1 to 27) indicated in the spectrum, refer to Table 1.

^1^H NMR spectra of liver extracts were inspected to investigate the characteristic resonances and gain an overview of the polar and non-polar compounds of pig liver. The complete list of putatively annotated metabolites based on the chemical shifts, signal multiplicity and chemical formula is highlighted in Table 1 and Table 2 for polar and non-polar fractions, respectively. A representative 600 MHz ^1^H NMR spectrum of the hepatic polar extract is shown in Figure 2. 

**Figure 3:**
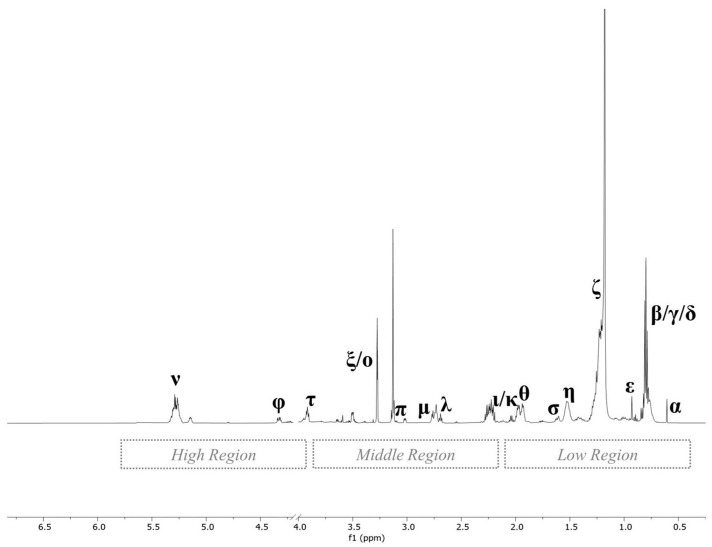
Representative 600 MHz 1D ^1^H NMR spectrum of the non-polar extract of pig liver. For the signal notation (α to φ) indicated in the spectrum, refer to Table 2.

Although the overlapping of some signals was observed, several metabolites belonging to different biochemical categories were identified along the region of interest (0–9 ppm). As can be seen, the spectrum of the polar extract was divided into three regions—aliphatic, middle and aromatic—described as follows. In the aliphatic region, the assignment of resonances at lower frequencies was easily conducted, since the signals and multiplicities (doublet, triplet, singlet) were well distinguishable. In the region from δ 0.9 to 2.7 ppm, the resonances were mainly attributable to amino acids (AA) and organic acids. Specifically, essential AA (isoleucine, leucine, valine, methionine), non-essential AA (alanine, proline, glutamate, tyrosine) and organic acids (lactate, acetate and succinate) were identified. The identification in the middle region of the ^1^H NMR polar spectrum from δ 3.0 to 5.2 ppm was very challenging due to the presence of many signals and the overlapping of peaks belonging to protons of different molecules. This region was mainly dominated by peaks attributable to carbohydrates (α-glucose), but choline and its derivatives (glycerophosphocholine and phosphorylcholine) were identified, too. The aromatic region from δ 6.5 to 8.5 ppm was mainly characterized by aromatic AA phenylalanine and tyrosine. Figure 3 presents a representative ^1^H NMR spectrum of the non-polar fraction of liver, which appeared less complex than that of the polar fraction, at a glance (Figure 2). 

Three main regions were identified: low-, middle- and high-frequency region. Low chemical shift (δ 0.6–2.2 ppm) was predominantly characterized by the intrachain proton of fatty acids and total cholesterol containing the sum of low-density lipoprotein (LDL) cholesterol, high-density lipoprotein (HDL) cholesterol, and very low-density lipoprotein (VLDL) cholesterol. In the middle and high frequencies (δ > 2.2 ppm), the spectrum was characterized by peaks of protons mainly attributable to phospholipids.

### 3.2. Data Exploration

**Figure 4:**
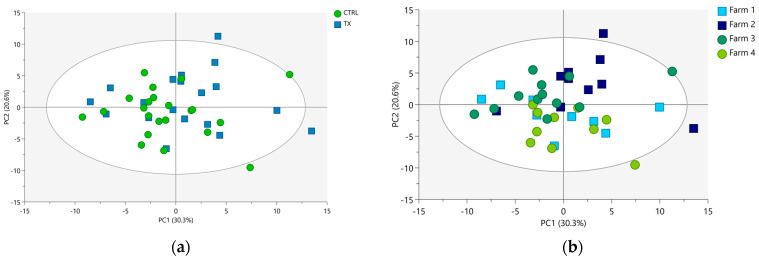
PCA score plots of pig liver polar extracts (R^2^X = 0.712; Q^2^ = 0.512) colored according to (**a**) group ID (CTRL, green circles; and TX, blue squares) and (**b**) Farm ID (Farm 1, blue squares; Farm 2, light blue squares; Farm 3, green circles; Farm 4, light green circles). The ellipse identifies the 95% confidence interval for Hotelling’s T2.

Unsupervised PCA was initially used as a preliminary tool to examine the structure and characteristics of both polar and non-polar extracts under investigation. For the polar fraction, 71.2% of the overall variance in the spectra was explained by a total of six extracted PCs, and the prediction ability was found to be around 51.2%. The two-dimensional score plot for PC1 and PC2 is reported in Figure 4a, where the samples are highlighted by group ID (TX and CTRL), and in Figure 4b, where the samples are colored according to Farm ID. Although the quality of the model was good, weak grouping behaviors emerged in samples based on group ID (Figure 4a) and Farm ID (Figure 4b), indicating a high within-groups variability existing in them.

**Figure 5:**
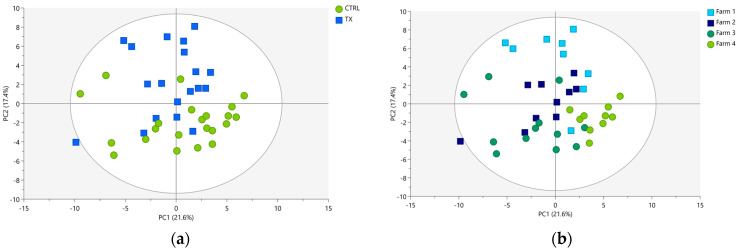
PCA score plots of pig liver non-polar extracts (R^2^X = 0.725; Q^2^ = 0.419) colored according to (**a**) group ID (CTRL, green circles; and TX, blue squares) and (**b**) Farm ID (Farm 1, blue squares; Farm 2, light blue squares; Farm 3, green circles; Farm 4, light green circles). The ellipse identifies the 95% confidence interval for Hotelling’s T2.

For the non-polar fraction, a total of six PCs covering 72.5% of the total variance were extracted. The goodness of fit and the prediction ability of this PCA model were similar to those provided by the PCA model of the polar fraction. Figure 5 shows the score plot for PC1 and PC2 of the non-polar extracts of liver samples colored based on group ID (CTRL and TX; Figure 5a) and Farm ID (Figure 5b).

In the non-polar extracts, a slightly higher grouping trend was observed compared to the polar extracts. Indeed, PC2 seems to have an influence on discriminating CTRL samples (PC2 negative values) from TX samples (PC2 positive values) (Figure 5a).

As illustrated in Figure 4 (PCA score plots of polar extracts) and Figure 5 (PCA score plots of non-polar extracts), the first two PCs collectively accounted for 50% and 39% of the overall extracted variability. The portion of the variability in the data matrices remaining unexplained by PC1 and PC2 alone should not be interpreted negatively for several reasons. Firstly, it is crucial to emphasize that the PCA was conducted using cross-validation to prevent overfitting. Consequently, the optimal number of PCs best suited for capturing the underlying data structure and the fraction of variance extracted by each of these components were automatically determined. As a result of cross-validation, the final PCA models for both polar and non-polar extracts were not limited to only two PCs but included six PCs, collectively explaining 71.2% and 72.5% of the total dataset variability. It is worth mentioning that the first two PCs, when considered individually, explain approximately ¾ of the overall variability for the polar extract (accounting for 70% (50% of a total of 71.2% in Figure 4)) and half of the overall variability (54% (39% of a total of 72.5% in Figure 5)) for the organic extract. This scenario is frequently encountered when applying multivariate statistical techniques in the analysis of high-dimensional omics data. The underlying factors contributing to the suboptimal cumulative score (PC1 + PC2) of explained variance may have included the natural complexity and high dimensionality of the data matrices. The dataset under analysis was highly complex and given the considerable number of original NMR variables subjected to PCA (i.e., 84 and 76 ppm buckets for polar and non-polar extract data matrices, respectively), reducing them to only two PCs, which capture almost all the variability, was highly unlikely. Another factor worthy of consideration is possible data noise. As is typical with omics data, the analyzed dataset inevitably contained natural variability unrelated to the primary factors of interest and linked to the high biological variability of the samples. Thus, this noise may have reduced the proportion of variability explained by the first two PCs.

Considering the known high biological variability of the samples under investigation, this finding was deemed promising. Therefore, the ability of supervised multivariate analysis to discriminate liver samples was investigated. 

### 3.3. Discriminant Models and Feature Selection

**Figure 6:**
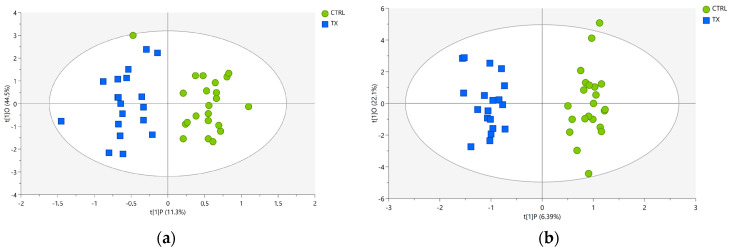
OPLS-DA score plots of pig liver (**a**) polar extracts and (**b**) non-polar extracts for the CTRL (green circles) and TX (blue squares) groups. The ellipse identifies the 95% confidence interval for Hotelling’s T2.

Following the encouraging outputs from the PCA, the supervised OPLS-DA was applied as a discriminant method able to distinguish the variation in the dataset with the predictive component, which is related to the class membership, and the orthogonal component describing the systematic variation within a class [44]. Two OPLS-DA models were built separately for polar and non-polar fractions aimed at distinguishing samples based on whether or not they were exposed to antibiotics and highlighting the driving forces among the variables most effective in discriminating between treated vs. untreated pigs. The unsupervised clustering trend became more pronounced, and the models’ diagnostic tools revealed extremely good performances of the models of both fractions, particularly in the case of the non-polar liver extract. The score scatter plots of the polar and non-polar extracts are depicted in Figure 6a and Figure 6b, respectively.

For the liver polar fraction, the two groups of samples were perfectly separated in the bi-dimensional space, since samples from the CTRL group were distributed along the positive t[1]P component, and samples from the TX group were distributed along the negative component. Nevertheless, a mild degree of heterogeneity was observed within samples in the same group, which were not closely grouped along the vertical axis. The intra-class non-predictive variability, of which 44.5% was collected by the t[1]O component, was expected to be present but did not hinder the correct discrimination of untreated (CTRL group) vs. treated (TX group) pigs, except for one sample in the CTRL group, which fell into the left side of the plot, corresponding to the negative scores, where all the TX samples were located, but within the 95% confidence ellipse (Figure 6a). Satisfying statistical metrics were provided by internal cross-validation of the model: a fitting ability of about 89% (R^2^X= 0.892), extracted predictive variation explaining around 77% of the information related to the specific class membership of the samples (R^2^Y = 0.774) and a predictive power of approximately 61% (Q^2^ = 0.613) proved the validity of the model. A classification accuracy of approximately 98% was obtained in the misclassification test, corresponding to only one sample being misclassified in the CTRL group.

Similar to the polar fraction, the OPLS-DA applied to the non-polar dataset returned very good quality parameters of the model: R^2^X = 0.939, R^2^Y = 0.948 and Q^2^ = 0.869. A correct classification rate of 100% was obtained, revealing that all samples were correctly allocated in their group ID, as can be observed by the perfect separation between the two groups in the score scatter plot reported in Figure 6b.

Subsequently, the VIP analysis was applied in order to identify the NMR spectral signals, which mostly contributed to the successful separation of the liver samples achieved by both OPLS-DA models. Overall, 17 and 11 NMR signals were found to be characterized with VIP values ≥ 1 in the polar and non-polar extracts, respectively. According to the results of the Mann–Whitney test and the two-sample *t*-test, 11 out of 17 selected features of the polar fraction and 6 out of 11 features of the non-polar fraction were statistically significantly different (*p* ≤ 0.05).

Following the assignment of the resonances, the list of the identified metabolites, which were significantly different (*p* ≤ 0.05) among the CTRL and TX groups—provided with their corresponding VIP score and FC value—was compiled and is reported in Table 3. As can be observed, unlike the NMR spectra of the polar extracts, in which the assignments of H resonances were unambiguously attributed to specific molecules, the NMR spectra of non-polar extracts were in some cases ascribed to classes of molecules (i.e., unsaturated fatty acids). According to the positive values of FC, all metabolites from the polar fraction appeared to be up-accumulated in the TX group of antibiotic treated pigs, while for the non-polar fraction, differences in metabolite accumulation were found.

## 4. Discussion

As far as the authors know, this is the first NMR-based untargeted metabolomics study comparing the liver metabolome of antibiotic-treated vs. untreated heavy pigs. An NMR-based analysis was used by Merrifield et al. [34] to provide a system overview of porcine metabolism via characterization of the liver and other organs and biofluid metabolomes to establish a metabolic framework from which pathology-or nutrition-based variations could be compared. In all living organisms, the liver is the organ in charge of the metabolism of major constituents (i.e., carbohydrates, proteins and lipids), but it is also recognized as the most important site for metabolic clearance of xenobiotics, synthesis of molecules and vitamin storage, playing a crucial role in metabolic homeostasis [45]. Based on the findings of the present pilot study, the application of an untargeted metabolomics approach returned different metabolic fingerprinting of pigs’ livers exposed to antimicrobial agents compared to untreated ones. Moreover, changes in the metabolism of the liver attributable to the differences in metabolite accumulation between TX and CTRL groups were observed, answering the starting biological question: “*Are there any metabolic differences between treated* vs. *untreated pigs?*”.

In the ^1^H NMR spectra of highly complex biological tissues, peak overlapping inevitably occurs, in particular for intact tissue sample analysis. In this case, the spectral assignment may be supported by 2D experiments [34]. In the present study, the polar and non-polar extracts of the liver were investigated separately, and 2D NMR experiments were not performed, as the resulting spectral signals were sufficiently resolved to enable the discrimination of resonances from the various compounds in both liver fractions. The investigation of separated aqueous and organic extracts of the liver is the approach commonly used in ^1^H NMR-based metabolomics studies aimed at investigating hepatic metabolic changes; this strategy was used to study paracetamol-induced hepatotoxicity in pigs [36], hyperlipidemic hamsters [38], chicken and avian metabolism [37,39] and the impact of dietary sesamin on Atlantic salmon [33]. The metabolic atlas of porcine liver provided by Merrifield et al. [34] was particularly useful for the spectra signal assignment of the polar extract, whereas the assignment of the non-polar extract was mainly supported by studies addressing the lipophilic extract of the liver [33,38,39], the review by Li et al. [33], as well as online public databases [40,41].

Relevant ^1^H NMR signals reported in Table 3 were investigated based on their fold change ratio to opportunely check the nature of metabolites characterizing the discrimination between the two groups. In particular, the up-accumulation of glucose (FC = 1.80), glutathione (FC = 1.10), proline (FC = 1.13), tryptophan (FC = 1.13), choline (FC = 1.23) and lactate (FC = 1.20) recorded in the polar liver extract from pigs in the TX group is probably due to a wide array of factors, among which, the antimicrobial administration and all related life conditions in which the pigs lived. It is worth mentioning that the antibiotic treatment characterizing pigs in the TX group most likely underlies a pathology, which has been cured, implying that the animal has experienced a physiological state different from that of a healthy animal. As a general remark, antimicrobial administration should occur in the presence of proven disease for responsible and prudent usage of antimicrobials, leading veterinarians to only prescribe them under strict conditions (i.e., clinical signs and symptoms, laboratory results, costs) [46]; considering this scenario, it cannot be excluded that pigs from the TX group might have experienced a stress condition during their life cycle, which, in other words, means that the sphere of animal welfare was threatened in the Health Domain—one Domain within the Five Domains model of animal welfare—and pigs were in a poor welfare condition for at least a well-defined time of their life (i.e., time of disease) [47]. In the field of animal-based measures, it is widely accepted that glucose represents the primary physiological indicator of stress [48]; during a stressful condition, the catecholamine released from the adrenal medulla regulates glycogenolysis in the liver, leading to maintenance of adequate glucose levels in blood circulation, entailing degradation of glycogen into glucose-6 phosphate, which is then hydrolyzed into glucose [49,50]. In addition to glycogenolysis, hepatic glucose production also relies on gluconeogenesis using glucose precursor molecules, such as amino acids, lactate, pyruvate and glycerol, for de novo synthesis of glucose [50]. The up-accumulation of lactate in the liver of pigs in the TX group highly supports the link between carbohydrate metabolism and animals living in a poor welfare condition, which may have been represented by the state of disease. In the present study, pigs in the TX group had 1.2 times as much lactate as those in the CTRL group, suggesting that increased energy demand characterized the pigs exposed to antibiotics administration. In fact, lactate is highly involved in the Krebs cycle of generating energy from stored energy reserves [51], representing an alarm bell for metabolic disorders in a living organism [35,52]. In this context, the authors refer to the higher energy state of the TX group, in which the disease status may have solicited a higher energy request from the diseased organism than in the healthy animals. In particular, in the pathological state, the greater demand for energy for the immune and reparative processes takes place in the face of a reduced ingestion of food and often a lower oxygen saturation, which characterizes the frequent diseases affecting the respiratory system of the pig. Glutathione (GSH) is a tripeptide, particularly concentrated in the liver, playing a key role in the defense against oxidative stress. Liver is the organ most vulnerable to toxins and oxidative stress, and the concentration of GSH is greatly sensitive to environmental factors, heavy metals, glucose and xenobiotics [53]. In this study, the accumulation of GSH was slightly higher in the TX group than in the CTRL group, and we may suppose that a role could be played by the disease state, together with the antibiotic treatment. However, the literature investigating the relationship between GSH and antibiotic treatment in the liver of pigs is poorly explored; therefore, only an assumption related to the biological role of metabolite in the investigated organ may be reported. Choline plays a key role in membrane integrity, lipid metabolism and methylation. Additionally, in this case, this metabolite was up-accumulated in the livers of pigs in the TX group. Similar to GSH, the literature is lacking for comparative purposes; however, the up-accumulation of choline seemed to follow the same direction as phosphatidylcholine—highly involved in regulating lipid, lipoprotein and energy metabolism [54]—whose accumulation was observed by assessing the non-polar extract of the liver. Proline, together with tryptophan, both up-accumulated in the TX group, are included in Aminoacyl-tRNA biosynthesis according to the Kyoto Encyclopedia of Genes and Genomes (KEGG) [55]. This finding may be supported by the fact that most antibiotics are designed to specifically target ribosomal protein synthesis and aminoacyl-tRNA synthetases—a family of enzymes playing a central role in protein synthesis [56]. Intriguing findings were obtained when considering the non-polar fraction of the liver. In fact, the fold change in the TX pigs’ group showed the down-accumulation of fatty acid residues ((CH_2_)n in fatty acyl chain, FC = 0.95), total cholesterol (C_18_H_3_ total cholesterol, FC = 0.89) and unsaturated fatty acid (–CHCH_2_CH = in fatty acyl chain:20:4/22:6, FC = 0.88); however, phospholipids (>C_3_H_2_ in the glycerol backbone of PL, FC = 1.52) and phosphatidylcholine (–CH_2_N^+^(CH_3_)_3_ in the PC head group, FC = 1.43) were up-accumulated in the TX pigs’ group. In addition to liver metabolome, the transcriptomic analysis provided insights for the understanding of enzyme regulation in the case of antimicrobial treatment. According to Hu et al. (2020), the transcriptome and DNA methylome analysis of pigs’ liver fed with low-dose of antibiotics displayed an increase in nicotinamide N-methyltransferase (NNMT) expression, a positive regulator of gluconeogenesis in primary hepatocytes, which could stabilize the Sirtuin 1 protein necessary for glucose and cholesterol metabolism to reduce the abundance of cholesterol in serum and liver [57,58]. The higher levels of glucose and the lower levels of cholesterol in the livers of pigs in the TX group could be supported by the role of NNMT, considering that liver is the organ characterized by the strongest expression of this enzyme [57,58]. Additionally, liver boasts of an excellent production of metabolic substrates for energy metabolism (i.e., fatty acids), which are made available during stressful situations to support the organism [49]. The proof of this might be found in the lower level of fatty acids in the liver characterizing the TX pigs’ group. Liver hepatocytes synthesize bile acids via cholesterol metabolism, and the decreasing level of total cholesterol (C_18_H_3_) observed in the TX group may suggest that high conversion activity in the liver is performed to promote the synthesis of bile acid. In addition, the up-accumulation of phosphatidylcholine in the TX group was found to be in agreement with the higher level of choline in the treated group, considering that choline undergoes phosphorylation in order to build the phospholipids.

## 5. Conclusions

The present study is the first step in broader research, which has the ultimate goal of identifying and validating biomarkers as authentication tools for antibiotic-free pork supply chains. As a first attempt to gain an insight into the metabolome of pigs, NMR spectroscopy was chosen as the instrumental analytical platform. This exploratory investigation demonstrated the feasibility of the NMR-based metabolomics approach for detecting metabolomic fingerprinting useful for the discrimination of livers of pigs on the basis of antibiotic treatment exposure. The untargeted approach was powerful in screening samples and detecting molecular signatures and changes in the liver metabolome, both polar metabolites and lipidome of pigs in the two considered conditions. This preliminary outcome encourages a more in-depth investigation via other analytical techniques widely used in metabolomics studies. Indeed, MS-based methods offer higher performance than NMR in terms of sensitivity—which is extremely useful for measuring species with low abundance but potentially valuable information—and specificity, helping the elucidation of the chemical structures of potential metabolites of interest.

This finding is very promising, taking into account the broad variability in the animals from commercial farms included in the study. Because the main objective of this pilot study was to ascertain the performances of an untargeted analytical strategy as a tool to answer the question, “Has this pig ever been treated with antibiotics?”, no specific focus was given to the classes of antibiotics the pigs were administered in the present study. The authors are of the opinion that a fit-for-purpose experimental design and the application of MS-based metabolomics platforms should enable the acquisition of more in-depth information and identification of putative biomarkers enabling the confirmation or rejection of an antibiotic treatment, bearing in mind that this issue serves the authenticity and health purposes in the pig chain.

## Figures and Tables

**Table 1:** Summary of the ^1^H NMR spectra signal assignment of the polar extract of pig liver.

Biochemical Category	N ^a^	Metabolite Name	Assignment	δ ppm and Multiplicity ^b^	Formula
AA and Derivatives	**1**	Isoleucine	γCH_3_	0.96 (t)	C_6_H_13_NO_2_
			δCH_3_	1.04 (d)	
			αCH	3.65 (d)	
	**2**	Leucine	δCH_3_	0.96 (t)	C_6_H_13_NO_2_
			βCH_2_	0.99 (d)	
			αCH	3.72 (m)	
			γCH	1.72 (m)	
	**3**	Valine	γCH_3_	0.98 (d)	C_5_H_11_NO_2_
			γCH_3_	1.04 (d)	
			βCH_2_	2.25 (m)	
			αCH	3.61(d)	
	**4**	Alanine	βCH_3_	1.49 (d)	C_3_H_7_NO_2_
	**5**	Lysine	δCH_2_	1.72 (m)	C_6_H_14_N_2_O_2_
	**8**	Taurine	N-CH_2_	3.25 (t)	C_2_H_7_NO_3_S
	**11**	Ornithine	½ γCH_2_	1.72 (m)	C_5_H_12_N_2_O_2_
			δCH_2_	3.04 (t)	
			αCH	3.77 (t)	
	**12**	Proline	γCH_2_	2.06 (m)	C_5_H_9_NO_2_
	**13**	Methionine	δCH_3_	2.13 (s)	C_5_H_11_NO_2_S
			αCH	3.78 (m)	
	**14**	Glutamate	βCH_2_	2.07 (m)	C_5_H_8_NO_4_^−^
				2.13 (m)	
			γCH_2_	2.35 (m)	
	**16**	Creatine	N-CH_3_	3.04 (s)	C_4_H_9_N_3_O_2_
	**18**	β-Alanine	CH_2_COOH	2.56 (t)	C_3_H_7_NO_2_
			N-CH_2_	3.18 (t)	
	**21**	Tyrosine	C3H&C5H	6.91 (d)	C_9_H_11_NO_3_
			C2H&C6H	7.20 (d)	
	**22**	Phenylalanine	C3H &C5H	7.33 (m)	C_9_H_11_NO_2_
			C4H	7.38 (m)	
			C2H&C6H	7.42 (m)	
	**24**	Threonine	γCH_3_	1.33 (d)	C_4_H_9_NO_3_
			αCH	3.57 (d)	
	**17**	Glutathione	αCH	3.77 (m)	C_10_H_17_N_3_O_6_S
	**23**	Tryptophan	αCH	4.06 (m)	C_11_H_12_N_2_O_2_
			C1H	7.33 (s)	
			C3H	7.55 (d)	
	**26**	Glutamine	βCH_2_	2.13 (m)	C_5_H_10_N_2_O_3_
			αCH	3.77 (t)	
Organic Acids	**4**	Lactate	βCH_3_	1.33 (d)	C_3_H_5_O_3_^−^
			αCH	4.12 (q)	
	**7**	Acetate	CH_3_	1.92 (s)	C_2_H_3_O_2_^−^
	**15**	Succinate	CH_2_	2.41 (s)	C_4_H_4_O_4_^−2^
Carbohydrates	**20**	Alpha-glucose	C_4_H	3.41 (t)	C_6_H_12_O_6_
			C_2_H	3.54 (m)	
			C_3_H	3.72 (t)	
			C_1_H	5.24 (d)	
		Beta-Glucose	C_2_H	3.24 (m)	
			C_5_H	3.83 (m)	
			C_6_H	3.90 (dd)	
			C_1_H	4.65 (d)	
Choline and Derivatives	**9**	Choline	N-(CH_3_)_3_	3.21 (s)	C_5_H_14_NO^+^
			βCH_2_	3.53 (dd)	
			αCH_2_	4.07 (m)	
	**10**	Glycerophosphocholine	N-(CH_3_)_3_	3.23 (s)	C_8_H_21_NO_6_P^+^
	**19**	Phosphorylcholine	N-(CH_3_)_3_	3.22 (s)	C_5_H_14_NO_4_P
Alcohol	**27**	Glycerol	CH	3.77 (m)	C_3_H_8_O_3_
			½ CH_2_	3.57 (m)	
			½ CH_2_	3.65 (m)	
Nucleoside	**25**	Adenosine	Ring protons	8.25 (s)	C_10_H_13_N_5_O_4_

^a^ N = numbering (1 to 27) of the signals indicated in Figure 2; ^b^ s = singlet; d = doublet; t = triplet; q = quartet; m = multiplet; dd = double doublets. All chemical shifts were verified, comparing experimental values with HMDB [41], BMRB [40] and the literature [33,34,37,38,39]. The IUPAC rules were employed for the molecule numbering system.

**Table 2:** Summary of the ^1^H NMR spectra signal assignment of the non-polar extract of pig liver.

Biochemical Category	N ^a^	Metabolite Name	Assignment	δ ppm and Multiplicity ^b^
Cholesterol	**α**	Total cholesterol	C_18_**H**_3_	0.61 (s)
	**β**		C_26_**H**_3_, C_27_**H**_3_	0.84 (d)
	**δ**		C_21_**H**_3_	0.82 (d)
	**ε**	Esterified cholesterol	C_19_**H**_3_	0.93 (s)
Fatty Acids	**γ**	Fatty acid residues	ꙍ-C**H**_3_	0.90 (t)
	**ζ**		(C**H**_2_)n	1.19 (m)
	**η**		COCH_2_–C**H**_2_	1.52 (m)
				1.61 (m)
	**θ**		C**H**_2_–CH=(MUFA and PUFA)	1.97 (m)
				2.04 (m)
	**κ**		–CO–C**H**_2_	2.23 (m)
	**λ**		–CH=CH–C**H**_2_–CH=CH– of linoleic acid	2.68 (t)
	**ν**		–C**H**=C**H**–	5.28 (m)
	**σ**		(–C**H**_2_ all FA except ARA and EPA)	1.61 (m)
	**μ**	FA, PUFA	CH=CH–C**H**_2_–(CH=CH–CH_2_)n	2.75 (m)
MAG and TAG	**ι**	Monoglycerides (MAG)	FA, RH–C**H**_2_–CO–O–C_2_	2.20 (m)
	**φ**	Triglycerides (TAG)	C_1_**H** and C_3_**H** of glycerol backbone	4.22 (dd)
			C_1_**H** and C_3_**H** of glycerol backbone	4.26 (dd)
Phospholipids	**ξ**	Sphingomyelin	(–CH_2_–N–(C**H**_3_)_3_) head group	3.27 (s)
	**ο**	Phosphatidylcholine (PC)	(–CH_2_–N–(C**H**_3_)_3_) head group	3.31 (s)
	**π**	Phosphatidylethanolamine (PE)	–CH_2_–C**H**_2_–NH_2_	3.13 (s)
	**τ**	Total phospholipids	Glycerol (C_3_**H**_2_) of phospholipids	3.92 (m) 4.02 (m)

^a^ N = notation (α to φ) of the signals indicated in Figure 3; ^b^ s = singlet; d= doublet; t = triplet; q = quartet; m = multiplet; dd = double doublets. All chemical shifts were verified, comparing experimental values with HMDB [41], BMRB [40] and the literature [33,35,38,39]. The IUPAC rules were employed for the molecule numbering system.

**Table 3:** Discriminant metabolites among the antibiotic-treated (TX group) and untreated pigs (CTRL group).

Metabolite	VIP Score ^a^	FC ^b^
Polar extract of pig liver		
Glucose	3.30	1.80
Glutathione	2.55	1.10
Proline	2.49	1.13
Tryptophan	2.29	1.13
Choline	1.30	1.23
Lactate	1.20	1.20
Non-polar extract of pig liver		
Fatty Acid	4.50	0.95
Phospholipids	2.92	1.52
Phosphatidylcholine	1.94	1.25
Total Cholesterol	2.88	0.89
Unsaturated Fatty Acid	1.08	0.88

^a^ VIP = variable importance in projection; ^b^ FC = fold change.

## Data Availability

The data used to support the findings of this study can be made available by the corresponding author upon request.
